# Physical Problems, Fatigue, and Healthy Lifestyle Behaviors Experienced by Women in the First 48 Hours of Postpartum Period

**DOI:** 10.1111/nhs.70113

**Published:** 2025-04-14

**Authors:** Yasemin Erkal Aksoy, Bihter Akin, Sema Dereli Yilmaz

**Affiliations:** ^1^ Department of Midwifery Health Sciences Faculty of Selcuk University Konya Türkiye

**Keywords:** behavior, fatigue, healthy, lifestyle, pain, postpartum

## Abstract

The postpartum period is a crucial phase in women's lives, characterized by physical challenges, fatigue, and lifestyle behaviors. This descriptive study aimed to assess physical challenges, fatigue, and healthy lifestyle behaviors in women during the first 48 h postpartum. Data were collected from 548 women hospitalized in the postpartum service of Meram Medical Faculty at Necmettin Erbakan University between December 2021 and August 2022, using a convenience sampling method. Data collection tools included the Personal Information Form, Postpartum Assessment Form, Fatigue Assessment Scale, and Healthy Lifestyle Behavior‐II Scale. Linear regression analysis (ENTER model) was conducted for advanced statistical evaluation. The study found that women employed outside the home (*p* = 0.001) and those with a high monthly income (*p* = 0.008) had higher levels of fatigue, while their levels of healthy lifestyle behaviors were also higher (*p* < 0.001, *p* = 0.002) compared to other groups. Breastfeeding challenges were associated with increased nipple pain (*p* = 0.002), incision pain (*p* = 0.003), and back pain (*p* = 0.012). Women accompanied by a partner reported higher fatigue levels (*p* = 0.005). Fatigue levels were affected by employment, cesarean section, breastfeeding experience, and incomplete areola compression by the baby.

**Summary**

- Several factors influence women's fatigue levels, including employment outside the home, having a cesarean delivery, experiencing challenges while breastfeeding, and the baby's inability to achieve full compression of the areola. Additionally, women who were accompanied by their partners reported higher levels of fatigue than those who were accompanied by their mothers or mothers‐in‐law.
- The HLSB‐II levels of women aged 31–49 years, who had graduated from college, were employed outside the home, and experienced no pregnancy‐related challenges, were higher than those in other groups.
- Women experiencing their first pregnancy reported higher levels of nipple pain, back pain, and waist pain compared to those who had two or more pregnancies.

## 1 Introduction

In the postpartum period, many of the mother's physiological systems undergo a process of physical adaptation. The reproductive, circulatory, respiratory, and musculoskeletal systems gradually return to their normal pre‐pregnancy functions (Nagtalon‐Ramos and Convery 2023). Breastfeeding is the first behavior that the mother begins learning and practicing upon holding the baby in the postpartum period. In the early postpartum period, the oxytocin hormone secreted from the mother via breastfeeding accelerates the involution process by making the uterus contract (Karaçay Yıkar et al. 2022; Stuebe et al. 2018). Mothers' physical and emotional needs for self‐care increase in the early postnatal days; thus, mothers may experience some physical and emotional discomfort (Aktaş and Karaçam 2017; Lambermon et al. 2020). Increased uterine contractions due to breastfeeding may cause abdominal pain in the mother during the early postpartum period. The mother may experience difficulty moving if an episiotomy or cesarean section (C‐section) has been performed. Furthermore, the mother may feel pain in the abdomen or perineum due to factors such as increased breast mass and carrying the baby on her lap (Cooklin et al. 2018; Jackson et al. 2019; Puritz et al. 2022). During the postpartum period, cultural expectations regarding mothers' self‐care and newborn care may vary significantly. While some cultures expect women to resume various tasks, such as caring for themselves and their babies, others may impose restrictions on postpartum activities. Both type of expectations may contribute to postpartum fatigue and challenges as mothers adjust to their new roles (Lambermon et al. 2020; O'Connor et al. 2018). Several studies have reported that the percentage of mothers experiencing fatigue during the postpartum period ranges from 18% to 82%, indicating significant variability in the prevalence of postpartum fatigue among different populations (Iwata et al. 2018; Khayamim et al. 2016). In a study conducted in Türkiye, the rate of fatigue at the 24th hour postpartum was 88.5% in mothers who gave birth vaginally and 92.7% in mothers who gave birth by cesarean section (Kilic et al. 2015). The fact that fatigue levels increase during the postpartum period may lead to multiple challenges, including difficulties with breastfeeding, sleep disturbances, and stress. Factors such as increased hemorrhages in the early postpartum period, length of hospital stay, first pregnancy, and inadequate social support are among the leading effects bringing on fatigue (Henderson et al. 2019; Kilic et al. 2015; Qian et al. 2021). All of these factors may adversely affect the health of both the mother and the baby. Many guidelines emphasize the importance of prenatal and early postpartum care to minimize postpartum morbidity and improve the quality of care (American Academy of Pediatrics (AAP) and American College of Obstetricians and Gynecologists (ACOG) Committee 2023; WHO 2022). The use of the Healthy Lifestyle Behavior‐II Scale (HLSB‐II) in the postpartum period has a significant place in protecting and improving the health of the mother and baby. A physically active lifestyle by mothers in the prenatal and postpartum period affects the healing process positively. A healthy and well‐planned diet increases the quality and quantity of the milk secreted by the mother, and it becomes easier for the mother to reach her ideal weight (Kahyaoğlu Süt and Hür 2020; Lim et al. 2020). The first 48 h of postpartum are important for early identification of problems and for planning interventions or late postpartum visits. Based on the literature, the number of studies evaluating the physical problems, fatigue, and HLSB‐II levels experienced by women in the postpartum period is limited (Aktaş and Karaçam 2017; Kahyaoğlu Süt and Hür 2020; Khayamim et al. 2016). This study contributes to the literature by examining the relationship between physical challenges, fatigue, and HLSB‐II levels during the critical first 48 h postpartum. It aims to provide a clearer understanding of how these factors interact and influence maternal well‐being in the early postpartum period, particularly in the context of Turkish healthcare practices. In this respect, the study is considered a contribution to the literature. Therefore, the study was designed to take a snapshot and evaluate the physical challenges, the fatigue, and the HLSB‐II levels experienced by women in the first 48 h postpartum.

## 2 Method

### 2.1 Objective

The aim of this study was to examine the physical challenges, fatigue, and levels of HLSB‐II experienced by women in the first 48 h of the postpartum period.

### 2.2 Study Design

In the study with a descriptive design, while the independent variables were determined as the sociodemographic characteristics and physical problems experienced by the women in the postpartum period, the dependent variables of the study were considered fatigue and HLSB‐II levels.

### 2.3 Research Questions

- Are fatigue and HLSB‐II levels of the women in the first 48 h of the postpartum period different in terms of the descriptive and obstetric characteristics?
- Are the levels of fatigue and HLSB‐II experienced by women in the first 48 h of the postpartum period different in terms of the breastfeeding characteristics and physical challenges?
- Is there a difference in terms of pain levels under the descriptive and obstetric characteristics of the women in the first 48 h of the postpartum period?
- What are the factors affecting the levels of fatigue and HLBS‐II in women in the first 48 h of the postpartum period?

### 2.4 Inclusion Criteria

The women 18 ≥ years, within 48 h of the postpartum period and agreeing voluntarily to participate in the study, and those with at least primary school degrees and without any psychological disorder (diagnosed as bipolar and related disorders, anxiety disorders, post‐traumatic stress disorder, etc.) were included in the study. Of the participants, those whose babies were in the neonatal intensive care unit (ICU), those who had postpartum complications (hemorrhage, sepsis, etc.), and those who did not complete all the questions on the data collection forms were excluded from the study.

### 2.5 Participants

The universe of the study was composed of the women in the first 48 h of the postpartum period accepting to participate in the study. The study was conducted between December 2021 and August 2022 on women hospitalized in the postpartum service of Meram Medical Faculty of Necmettin Erbakan University, affiliated with the Ministry of Health, in Konya/Türkiye. The sample size was determined using the G*Power 3.1.7 program, based on the known average scores of the Fatigue Assessment Scale (FAS) and the HLSB‐II, and a higher sample size was obtained with the known average score of the HLSB‐II. The potential risk of sample size bias was thought to be eliminated by this calculation. With the use of the known average score of HLSB‐II (131.53 ± 22.13), therefore, the sample size was determined as 546 with an effect size of 0.09 and 80% power within one‐point deviation (Erkal Aksoy and Özentürk 2021). Considering the data losses in the study, 570 women in the postpartum period were interviewed, and 10 whose babies were in the neonatal ICU and 12 failing to fill out the forms completely were not included in the study. Therefore, the study was completed with 548 women.

### 2.6 Data Collection

Participants were recruited using the convenience sampling method, and data were collected by self‐report. Each participant spent an average of 20–25 min completing the data collection form. During the study, they remained in postpartum service rooms with either single or double beds. To ensure a quiet environment and minimize external distractions, the researcher requested accompanying persons to leave the room during data collection. Participants were allowed to pause completing the form for personal needs, such as breastfeeding or using the restroom, before resuming.

### 2.7 Data Collection Tools

As data collection tools, the Personal Information Form (PIF), Postpartum Assessment Form (PAF), FAS, and HLSB‐II Scale were used to evaluate the levels of physical challenges, fatigue, and healthy behaviors experienced by women in the postpartum period.

#### 2.7.1 PIF

The PIF was prepared by the researchers through a literature review and consists of 19 questions that focus on the sociodemographic (age, educational status, type of family, etc.) and obstetric (gravida, number of deliveries, mode of delivery, parity, and complications during childbirth etc.) characteristics of women (Aktaş and Karaçam 2017; Kahyaoğlu Süt and Hür 2020).

#### 2.7.2 PAF

Including the information about the physical challenges experienced by women in the postpartum period and breastfeeding, PAF consists of 12 questions. In the study, the vertical form of the visual pain scale was used to evaluate the severity of pain in areas, such as the breast, back, and waist. At the margins of the scale, there are lines divided into 10 equal parts expressing “no pain (0)” on one margin and “severe pain (10)” on the other (Kilic et al. 2015; Selçuk and Saydam 2019).

#### 2.7.3 FAS

Consists of 10 items, the scale was developed by Michielsen et al. in 2003, and the Turkish version was created by Selcuk and Karaca Saydam in 2019. As a one‐dimensional scale, FAS consists of the items evaluating generally the physical and cognitive aspects of the individual and questioning how s/he feels. Of those 10 items, while eight (1, 2, 3, 5, 6, 7, 8, 9) reflect the negative aspects of fatigue, the remaining two items (4 and 10) contain retrospective data revealing the energy status of the participants. In scoring, these two items should be reversed. The scale is a 5‐point Likert‐type instrument, with response options ranging from 1 (never) to 5 (always): 1 = never, 2 = sometimes, 3 = regularly, 4 = often, and 5 = always. The total score ranges from a minimum of 10 to a maximum of 50, with higher scores indicating increased fatigue symptoms. The Cronbach Alpha coefficient obtained in the original scale was 0.90, and the coefficient was 0.70 in the study by Selcuk and Saydam (Michielsen et al. 2003; Selçuk and Saydam 2019). In this study, however, the Cronbach Alpha coefficient of the scale was determined to be 0.84.

#### 2.7.4 HLSB‐II

Walker et al. developed a scale to evaluate the health‐promoting behaviors concerning the healthy lifestyle of an individual in 1987 (Walker et al. 1987), and the validity and reliability of the scale were conducted and put into Turkish by Bahar et al. in 2008 (Bahar et al. 2008). HLSB‐II is composed of 52 items, all investigating the positive health‐promoting behaviors, and has four options scored as never (1), sometimes (2), often (3), and regularly (4). While the lowest score obtained from the whole scale is 52, the highest score is 208, and the total score determines the score of healthy lifestyle behaviors. Additionally, the scale is composed of six subdimensions, such as health responsibility, physical activity, nutrition, spiritual development, interpersonal relations, and stress management (Bahar et al. 2008; Walker et al. 1987). The Cronbach Alpha coefficient was calculated as 0.92 in both the original version (Walker et al. 1987) and the validity and reliability study (Bahar et al. 2008), and this study.

### 2.8 Statistical Analysis

The analysis of the data obtained from the study was evaluated via the computer using the Statistical Package for Social Sciences for Windows, version 20.0 (IBM Corp. SPSS Statistics 2017). Whether the data showed normal distribution or not was checked with the Kolmogorov–Smirnov test, and the descriptive statistics were given as numbers, percentages, mean, and standard deviation (SD). While the difference between the scale scores and the bivariate groups was evaluated using the *t*‐test for normally distributed data in independent groups, the Mann–Whitney U test was used to assess the non‐normally distributed data. Additionally, the difference between the three‐group variables and the scale scores was analyzed by the Analysis of Variance test (ANOVA). For advanced statistical evaluation, the ENTER model of linear regression analysis was used. Results were considered statistically significant at a *p* value of less than 0.05.

## 3 Results

The mean gestational age of the participants was 38.75 ± 1.65 weeks (min = 29, max = 42). The mean scores the participants obtained from the overall FAS and HLSB‐II were 23.07 ± 6.72 and 135.62 ± 21.49, respectively. The mean level of nipple pain was 3.00 ± 2.68, and the mean level of C‐section or episiotomy incision site pain was 4.59 ± 3.10. The mean scores for back pain and waist pain were 3.82 ± 2.97 and 3.96 ± 3.06, respectively.

In this study, the mean scores of the FAS level, the HLSB‐II total, and all sub‐dimensions were significantly higher for women with employment outside the home than for those with no employment outside the home (*p* = 0.001). Additionally, the FAS levels were found to be statistically significantly higher in women who smoked (*p* = 0.036) and those who regularly used medications due to chronic diseases (*p* = 0.041) compared to non‐smokers and those not using medications. The averages of HLSB‐II total (*p* = 0.045) and physical activity (*p* < 0.001) and interpersonal relations (*p* = 0.002) sub‐dimension scores of the women experiencing challenges during pregnancy were determined to be low. Given the comparisons between the women given C‐section births and vaginal deliveries, the women having C‐section deliveries were determined to have higher FAS (*p* = 0.001) levels and lower levels of physical activity (*p* = 0.028) and spiritual development (*p* = 0.023) of the sub‐dimensions of HLSB‐II. The physical activity score of the HLSB‐II sub‐dimensions of the women undergoing episiotomy during the delivery was lower than those who did not (*p* = 0.003). The total HLSB‐II scores (*p* = 0.035) and the sub‐dimension scores for health responsibilities (*p* = 0.011), nutrition (*p* = 0.012), and interpersonal relations (*p* = 0.001) were significantly lower in women who experienced lacerations during delivery compared to those without lacerations.

The total HLSB‐II scores (*p* = 0.010) and the subdimension scores for physical activity (*p* = 0.049), nutrition (*p* < 0.001), spiritual development (*p* = 0.036), and stress management (*p* = 0.028) of the women breastfeeding were determined to be lower compared to those without breastfeeding. While the participants who breastfed their babies just after birth and within the first 30 min after the birth had higher average levels of physical activity than did those who started breastfeeding 1 h after the birth (*p* < 0.001). The participants who breastfed immediately after giving birth had higher average stress management scores than did those who breastfed 2 h or later (*p* = 0.028). The average FAS score of those experiencing challenges while breastfeeding was higher than that of those not experiencing breastfeeding problems (*p* = 0.001). The average total FAS score (*p* < 0.001), as well as the physical activity (*p* < 0.001) and nutrition (*p* = 0.024) subdimension scores of HLSB‐II, were found to be lower among women whose baby's mouth completely compressed the areola for effective milk ejection compared to those whose baby's mouth did not. The mean FAS score was higher for women who were accompanied by their partner than it was for those who were accompanied by their mother or mother‐in‐law (*p* = 0.005). The mean health responsibility (*p* < 0.001) and physical activity (*p* < 0.001) scores of the participants having a partner as a patient accompanist were higher than those of the others (Aunt, sister, etc.) (Table 1).

**TABLE 1:** Comparison of descriptive, obstetric, and breastfeeding characteristics with scale scores of women in the postpartum period.

				Subdimensions of HLSBS					
Variables	*n* (%)	FAS total	HLSBS total	Health responsibility	Physical activity	Nutrition	Spiritual development	Interpersonal relations	Stress management
		Mean ± SD	Mean ± SD	Mean ± SD	Mean ± SD	Mean ± SD	Mean ± SD	Mean ± SD	Mean ± SD
Age groups (Mean ± SD = 27.60 ± 5.42 (min = 18, max = 49))									
18–30 years	382 (69.7)	22.74 ± 6.64	134.01 ± 20.12	23.33 ± 4.73	16.09 ± 4.36	22.49 ± 4.21	26.30 ± 4.85	26.13 ± 4.86	19.65 ± 3.83
31–49 years	166 (30.3)	23.85 ± 6.85	139.34 ± 24.01	23.64 ± 5.66	17.18 ± 5.22	23.18 ± 4.36	27.39 ± 5.25	26.78 ± 5.23	21.16 ± 5.04
*t*		−1.787	−2.685	−0.622	−2.352	−1.724	−2.343	−1.404	−3.851
*p*		0.075	**0.013**	0.534	**0.019**	0.085	**0.019**	0.161	**< 0.001**
Educational status									
Primary^a^	134 (24.5)	24.08 ± 6.32	133.17 ± 20.48	22.88 ± 4.50	16.69 ± 4.54	22.77 ± 4.12	25.75 ± 5.27	25.41 ± 5.14	19.65 ± 4.23
Secondary^b^	314 (57.3)	22.82 ± 6.92	134.13 ± 21.30	23.21 ± 5.30	16.14 ± 4.61	22.46 ± 4.27	26.50 ± 4.88	25.94 ± 4.79	19.86 ± 4.20
College^c^	100 (18.2)	22.53 ± 6.52	143.61 ± 21.81	24.83 ± 4.57	16.94 ± 4.95	23.36 ± 4.42	28.24 ± 4.65	28.76 ± 4.63	21.48 ± 4.40
*F*		2.085	8.762	5.038	1.419	1.694	7.515	15.897	6.467
*p*		0.125	**0.001** ^**c>a,b**^	**0.007** ^**c>a,b**^	0.243	0.185	**0.001** ^**c>a,b**^	**< 0.001** ^**c>a,b**^	**0.002** ^**c>a,b**^
Type of family									
Nucleus	459 (83.8)	22.99 ± 6.82	136.50 ± 21.78	23.66 ± 5.08	16.35 ± 4.65	22.68 ± 4.36	26.99 ± 4.96	26.54 ± 5.00	20.26 ± 4.35
Extended	89 (16.2)	23.50 ± 6.18	131.08 ± 19.46	22.22 ± 4.56	16.75 ± 4.70	22.78 ± 3.77	24.77 ± 4.79	25.24 ± 4.72	19.30 ± 3.86
*t*		−0.654	2.356	2.661	−0.732	−0.198	3.881	2.249	2.109
*p*		0.513	**0.020**	**0.009**	0.465	0.843	**< 0.001**	**0.025**	**0.037**
Employment outside the home									
Yes	85 (15.5)	25.78 ± 8.51	149.69 ± 25.39	25.44 ± 4.81	20.25 ± 5.43	25.17 ± 5.31	28.27 ± 4.59	27.58 ± 5.47	22.95 ± 4.47
No	463 (84.5)	22.58 ± 6.22	133.04 ± 19.66	23.05 ± 4.98	15.71 ± 4.14	22.25 ± 3.88	26.33 ± 5.02	26.09 ± 4.85	19.58 ± 4.04
*t*		3.315	5.735	4.086	7.327	4.840	3.309	2.545	6.923
*p*		**0.001**	**< 0.001**	**< 0.001**	**< 0.001**	**< 0.001**	**0.001**	**0.011**	**< 0.001**
Monthly income level of family									
Income less than expenses^a^	73 (13.3)	24.28 ± 7.34	131.19 ± 16.54	21.21 ± 4.61	15.78 ± 4.22	22.50 ± 3.09	26.69 ± 4.62	25.54 ± 4.95	19.43 ± 3.41
Income equal to expenses^b^	423 (77.2)	22.60 ± 6.41	135.28 ± 21.34	23.60 ± 5.05	16.32 ± 4.56	22.55 ± 4.24	26.52 ± 5.03	26.25 ± 5.05	20.02 ± 4.28
Income more than expenses^c^	52 (9.5)	25.21 ± 7.70	144.63 ± 26.22	25.09 ± 4.48	18.07 ± 5.70	24.23 ± 5.55	27.40 ± 5.25	28.03 ± 4.06	21.78 ± 5.00
*F*		4.903	6.289	10.508	4.092	3.708	0.713	4.050	5.038
*p*		**0.008** ^**c>b**^	**0.002** ^**c>a,b**^	**< 0.001** ^**b,c>a**^	**0.017** ^**c>a,b**^	**0.025** ^**c>b**^	0.490	**0.018** ^**c>a,b**^	**0.007** ^**c>a,b**^
Classification of BMI (Mean ± SD = 27.76 ± 4.43 (min = 18.50, max = 44.63))									
Normal 18.50–24.99^a^	153 (27.9)	24.73 ± 6.81	136.63 ± 21.23	23.88 ± 4.55	16.98 ± 5.23	23.09 ± 4.50	26.50 ± 4.88	26.41 ± 4.69	19.75 ± 3.54
Overweight 25.00–29.99^b^	242 (44.2)	22.19 ± 6.41	133.75 ± 21.16	22.63 ± 5.18	16.15 ± 4.14	22.06 ± 4.14	26.57 ± 4.85	26.06 ± 5.18	20.27 ± 4.44
Obese ≥ 30.00^c^	153 (27.9)	22.81 ± 6.85	137.58 ± 22.16	24.22 ± 5.07	16.28 ± 4.81	23.32 ± 4.11	26.86 ± 5.36	26.67 ± 4.93	20.21 ± 4.69
*F*		7.008	1.725	5.674	1.598	5.027	0.240	0.733	0.754
*p*		**0.001** ^**a>b**^	1.729	**0.004** ^**a,c>b**^	0.203	**0.007** ^**c>b**^	0.786	0.481	0.471
Smoking status									
Yes	40 (7.3)	25.25 ± 6.64	142.05 ± 26.13	25.05 ± 4.54	19.80 ± 5.97	23.57 ± 5.60	26.25 ± 5.01	26.52 ± 4.78	20.85 ± 4.53
No	508 (92.7)	22.90 ± 6.70	135.12 ± 21.03	23.29 ± 5.04	16.15 ± 4.44	22.63 ± 4.14	26.66 ± 5.00	26.31 ± 5.00	20.05 ± 4.26
*t*		2.107	1.596	1.979	3.677	1.028	−0.368	0.270	1.076
*p*		**0.036**	0.118	**0.048**	**0.001**	0.310	0.713	0.788	0.282
Existence of chronic diseases									
Yes	34 (6.2)	23.79 ± 5.06	132.58 ± 17.83	23.00 ± 3.37	14.17 ± 3.10	22.38 ± 3.45	26.58 ± 4.86	26.70 ± 4.44	19.73 ± 4.78
No	514 (93.8)	23.03 ± 6.82	135.83 ± 21.71	23.45 ± 5.12	16.57 ± 4.71	22.72 ± 4.32	26.63 ± 5.01	26.30 ± 5.01	20.13 ± 4.25
*Z*		−1.092	−0.778	−0.165	−2.990	−0.351	−0.038	0.548	−1.111
*p*		0.275	0.437	0.869	**0.003**	0.726	0.970	0.584	0.266
Regular use of medications due to chronic diseases									
Yes	27 (4.9)	24.92 ± 5.12	127.55 ± 15.66	21.40 ± 3.43	12.55 ± 2.56	22.88 ± 3.72	26.07 ± 4.48	26.88 ± 4.71	17.74 ± 2.69
No	521 (95.1)	22.98 ± 6.78	136.04 ± 21.68	23.53 ± 5.07	16.62 ± 4.66	22.69 ± 4.30	26.66 ± 5.02	26.30 ± 4.99	20.23 ± 4.32
*Z*		−2.042	−1.893	−2.151	−4.823	−0.321	−0.367	−0.660	−2.956
*p*		**0.041**	0.058	**0.031**	**< 0.001**	0.748	0.713	0.510	**0.003**
Challenges experienced in pregnancy									
Yes	55 (10.0)	23.43 ± 5.88	130.12 ± 20.58	22.67 ± 4.15	14.18 ± 4.68	22.90 ± 4.36	26.96 ± 4.57	24.36 ± 4.96	19.03 ± 4.10
No	493 (90.0)	23.03 ± 6.81	136.24 ± 21.53	23.51 ± 5.11	16.67 ± 4.60	22.68 ± 4.26	26.59 ± 5.05	26.54 ± 4.94	20.23 ± 4.29
*t*		0.416	−2.007	−1.384	−3.800	−0.374	0.513	−3.111	−1.964
*p*		0.678	**0.045**	0.170	**< 0.001**	0.708	0.608	**0.002**	0.050
Gravidas									
One pregnancy	146 (26.6)	23.83 ± 6.60	135.35 ± 20.81	23.15 ± 4.70	16.28 ± 4.02	22.58 ± 4.56	26.22 ± 5.10	26.43 ± 4.87	20.66 ± 4.24
Pregnancies ≥ 2	402 (73.4)	22.80 ± 6.75	135.72 ± 21.76	23.52 ± 5.14	16.47 ± 4.88	22.74 ± 4.16	26.78 ± 4.96	26.29 ± 5.02	19.91 ± 4.29
*t*		1.591	−0.179	−0.756	−0.465	−0.365	−1.154	0.306	1.823
*p*		0.112	0.858	0.450	0.642	0.716	0.249	0.760	0.069
Number of deliveries									
Primiparous	200 (36.5)	24.14 ± 6.48	135.39 ± 20.63	23.40 ± 4.85	16.27 ± 3.98	22.44 ± 4.27	26.30 ± 5.02	26.64 ± 5.05	20.33 ± 4.02
Multiparous	348 (63.5)	22.46 ± 6.79	135.76 ± 22.00	23.44 ± 5.13	16.50 ± 5.02	22.85 ± 4.26	26.82 ± 4.98	26.15 ± 4.93	19.98 ± 4.43
*t*		2.833	−0.198	−0.095	−0.613	−1.078	−1.168	1.104	0.904
*p*		**0.005**	0.843	0.924	0.540	0.282	0.243	0.270	0.366
Mode of delivery									
Vaginal delivery	339 (61.9)	22.27 ± 6.27	135.31 ± 21.68	23.26 ± 4.90	16.76 ± 4.53	22.89 ± 4.25	26.25 ± 4.88	26.01 ± 5.10	20.11 ± 4.19
Cesarean section	209 (38.1)	24.38 ± 7.22	136.13 ± 21.23	23.68 ± 5.22	15.86 ± 4.82	22.39 ± 4.29	27.25 ± 5.13	26.84 ± 4.73	20.10 ± 4.45
*t*		−3.485	−0.435	−0.940	2.196	1.344	−2.281	−1.893	−0.046
*p*		**0.001**	0.664	0.348	**0.028**	0.180	**0.023**	0.059	0.963
Episiotomies performed during deliveries (*n* = 339)									
Yes	187 (55.2)	22.63 ± 5.77	134.11 ± 18.08	23.20 ± 4.36	16.09 ± 3.84	22.70 ± 3.90	26.16 ± 4.65	26.04 ± 5.13	19.89 ± 3.80
No	152 (44.8)	21.82 ± 6.82	136.79 ± 25.41	23.34 ± 5.50	17.58 ± 5.16	23.13 ± 4.64	26.36 ± 5.17	25.97 ± 5.08	20.39 ± 4.61
*t*		1.180	−1.096	−0.243	−2.952	−0.942	−0.367	−0.133	−1.096
*p*		0.239	0.274	0.808	**0.003**	0.347	0.714	0.894	0.274
Lacerations developing during deliveries (*n* = 339)									
Yes	160 (47.2)	22.58 ± 5.63	132.69 ± 21.02	22.56 ± 4.45	16.96 ± 4.68	22.28 ± 4.08	25.93 ± 4.86	25.02 ± 5.23	19.93 ± 4.13
No	179 (52.8)	22.00 ± 6.78	137.65 ± 22.05	23.89 ± 5.20	16.58 ± 4.40	23.44 ± 4.33	26.54 ± 4.90	26.89 ± 4.83	20.28 ± 4.24
*t*		0.852	−2.115	−2.547	0.761	−2.540	−1.149	−3.425	−0.775
*p*		0.395	**0.035**	**0.011**	0.447	**0.012**	0.251	**0.001**	0.439
Breastfeeding status									
Yes	525 (95.8)	23.04 ± 6.56	135.08 ± 21.28	23.36 ± 5.01	16.28 ± 4.50	22.55 ± 4.25	26.53 ± 4.98	26.31 ± 4.99	20.02 ± 4.25
No	23 (4.2)	23.95 ± 9.91	148.17 ± 23.04	24.78 ± 5.34	19.52 ± 6.90	26.08 ± 3.21	28.86 ± 4.87	26.73 ± 4.83	22.17 ± 4.76
*Z*		−0.237	−2.588	−0.983	−1.968	−4.178	−2.097	−0.552	−2.203
*p*		0.813	**0.010**	0.326	**0.049**	**< 0.001**	**0.036**	0.581	**0.028**
Breastfeeding times (*n* = 525)									
Just after birth^a^	171 (32.6)	23.46 ± 7.95	137.38 ± 22.85	23.62 ± 5.41	17.16 ± 4.45	22.93 ± 4.55	26.70 ± 5.29	26.23 ± 4.84	20.70 ± 4.84
In first 30 min after birth^b^	168 (32.0)	22.63 ± 6.70	136.23 ± 22.14	23.48 ± 5.26	16.85 ± 4.72	22.64 ± 4.54	26.63 ± 5.01	26.59 ± 4.80	20.01 ± 4.09
In first 1 h after birth^c^	123 (23.4)	22.91 ± 4.01	132.69 ± 18.29	23.37 ± 4.12	14.98 ± 3.50	21.90 ± 3.52	26.49 ± 4.62	26.38 ± 5.47	19.56 ± 3.56
In first ≥ 2 h after birth^d^	63 (12.0)	23.22 ± 6.00	130.39 ± 19.10	22.34 ± 4.74	14.92 ± 4.95	22.55 ± 3.82	25.88 ± 4.81	25.61 ± 4.92	19.06 ± 3.95
*F*		0.478	2.382	1.048	8.814	1.453	0.445	0.604	3.069
*p*		0.698	0.069	0.371	**< 0.001** ^**a,b>c,d**^	0.227	0.721	0.613	**0.028** ^**a>d**^
Status of experiencing challenges in breastfeeding (*n* = 525)									
Yes	81 (15.4)	25.66 ± 7.90	133.91 ± 22.28	22.44 ± 4.67	15.65 ± 5.00	22.79 ± 4.57	26.85 ± 5.14	26.18 ± 4.98	19.98 ± 4.44
No	444 (84.6)	22.56 ± 6.17	135.29 ± 21.11	23.53 ± 5.05	16.40 ± 4.40	22.51 ± 4.19	26.47 ± 4.96	26.33 ± 4.99	20.02 ± 4.21
*t*		3.355	−0.536	−1.806	−1.256	0.538	0.617	−0.249	−0.077
*p*		**0.001**	0.592	0.071	0.212	0.591	0.538	0.803	0.939
Existence of nipple fissure and hemorrhage (*n* = 525)									
Yes	110 (21.0)	23.31 ± 6.28	135.02 ± 19.62	23.67 ± 3.86	15.94 ± 4.01	22.70 ± 4.36	26.44 ± 4.80	26.67 ± 4.63	19.58 ± 3.91
No	415 (79.0)	22.96 ± 6.63	135.09 ± 21.72	23.28 ± 5.27	16.37 ± 4.62	22.51 ± 4.22	26.56 ± 5.04	26.21 ± 5.08	20.13 ± 4.33
*t*		0.500	−0.029	0.857	−0.891	0.424	−0.217	0.851	−1.219
*p*		0.617	0.977	0.393	0.373	0.672	0.829	0.395	0.223
Full compression of baby's mouth the areola (*n* = 525)									
Yes	418 (79.6)	22.40 ± 6.08	134.43 ± 20.39	23.46 ± 4.96	15.89 ± 4.29	22.31 ± 3.98	26.52 ± 4.91	26.29 ± 4.97	19.94 ± 4.18
No	107 (20.4)	25.50 ± 7.71	137.61 ± 24.39	23.00 ± 5.18	17.81 ± 4.96	23.51 ± 5.06	26.59 ± 5.30	26.36 ± 5.06	20.32 ± 4.49
*t*		−3.853	−1.244	0.850	−3.660	−2.281	−0.142	−0.121	−0.835
*p*		**< 0.001**	0.215	0.396	**< 0.001**	**0.024**	0.887	0.904	0.404
Existence of accompanist for postpartum women									
Yes	516 (94.2)	22.98 ± 6.80	135.65 ± 21.19	23.50 ± 5.01	16.28 ± 4.57	22.63 ± 4.21	26.72 ± 5.02	26.39 ± 4.99	20.10 ± 4.27
No	32 (5.8)	24.53 ± 5.06	135.28 ± 26.28	22.21 ± 5.25	18.59 ± 5.60	23.78 ± 5.00	25.15 ± 4.50	25.31 ± 4.68	20.21 ± 4.54
*t*		−1.260	0.094	1.402	−2.730	−1.471	1.901	1.191	−0.146
*p*		0.208	0.925	0.162	**0.007**	0.142	0.065	0.234	0.884
Accompanist with postpartum women (*n* = 516)									
Partner^a^	61 (11.8)	25.37 ± 8.10	138.93 ± 23.50	24.63 ± 4.76	18.21 ± 4.93	22.55 ± 5.04	26.16 ± 5.07	26.59 ± 4.16	20.77 ± 4.29
Mother^b^	255 (49.4)	22.67 ± 6.39	137.26 ± 21.44	24.12 ± 5.19	16.74 ± 4.37	22.51 ± 4.05	27.11 ± 5.24	26.54 ± 5.20	20.21 ± 4.20
Mother‐in‐law^c^	82 (15.9)	21.47 ± 5.77	133.67 ± 19.14	22.70 ± 4.87	15.64 ± 4.89	23.40 ± 4.19	25.89 ± 4.21	25.90 ± 5.05	20.12 ± 3.99
Others (aunt, sister, etc.)^d^	118 (21.5)	23.48 ± 7.30	131.83 ± 20.34	22.11 ± 4.46	14.74 ± 4.04	22.41 ± 4.09	26.75 ± 4.97	26.29 ± 4.91	19.51 ± 4.58
*F*		4.323	2.514	6.289	9.957	1.089	1.535	0.391	1.291
*p*		**0.005** ^**a>b,c**^	0.058	**< 0.001** ^**a,b>d**^	**< 0.001** ^**a>c,d**^	0.353	0.205	0.759	0.277

*Note:* FAS: Fatigue assessment scale, HLSBS: Healthy life style behavior scale, SD: Standard deviation, *t*: Independent groups *t*‐test, *Z*: Mann–Whitney U test, F: ANOVA test and post hoc Tukey test were used. In the table, *p* values < 0.05 are shown in bold and the source of the difference between the groups is shown with symbols a, b and c.

The factors affecting the pain levels in the nipples, C‐section or episiotomy incision site, and back and waist experienced by the women in the postpartum period were also investigated. Based on the age groups, the women between 31 and 49 years of age were determined to have higher pain levels on C‐section or episiotomy incision sites (*p* = 0.013), back (*p* = 0.006) and waist (*p* = 0.001), compared to those between the ages of 18 and 30. The women with the normal BMI values had higher pain levels on C‐section or episiotomy incision sites than the overweight and obese women (*p* = 0.035). The level of waist pain of the women experiencing challenges during pregnancy was higher than those experiencing no challenges (*p* < 0.001). It was determined that the women with the first pregnancy had higher nipple pain levels of pain in the nipple (*p* = 0.013), back (*p* = 0.009), and waist (*p* = 0.034) than those experiencing two or more pregnancies. The women undergoing C‐sections had a higher level of pain at the incision site than those with a vaginal delivery (*p* < 0.001). The participants experiencing challenges while breastfeeding had higher levels of pain in the nipple (*p* = 0.002), C‐section or episiotomy incision site (*p* = 0.003), and back (*p* = 0.012), compared to those experiencing no challenges in breastfeeding. The women whose baby's mouth compressed the areola fully for productive milk ejection had lower levels of pain in the nipple (*p* < 0.001), back (*p* < 0.001), and waist (*p* < 0.001), compared to those who did not (Table 2).

**TABLE 2:** Comparison of descriptive and obstetric characteristics, and pain levels of postpartum women.

Variables	Nipple pain	C‐section or episiotomy incision site pain	Back pain	Waist pain
	Mean ± SD	Mean ± SD	Mean ± SD	Mean ± SD
Age groups				
18–30 years	3.16 ± 2.61	4.38 ± 3.17	3.59 ± 2.92	3.68 ± 3.02
31–49 years	3.04 ± 2.80	5.07 ± 2.88	4.34 ± 3.04	4.60 ± 3.08
*t*	0.412	−2.495	−2.744	−3.232
*p*	0.681	**0.013**	**0.006**	**0.001**
Classification of BMI				
Normal (18.50–24.99)^a^	3.35 ± 2.86	5.13 ± 3.12	4.15 ± 2.95	4.27 ± 3.03
Overweight (25.00–29.99)^b^	2.97 ± 2.63	4.41 ± 3.06	3.82 ± 2.92	3.98 ± 3.05
Obese (≥ 30.00)^c^	2.69 ± 2.56	4.32 ± 3.08	3.49 ± 3.06	3.62 ± 3.10
*F*	2.292	3.364	1.849	1.714
*p*	0.102	**0.035** ^**a>b.c**^	0.158	0.181
Status of experiencing challenges in pregnancy				
Yes	2.50 ± 2.64	4.90 ± 2.30	4.38 ± 3.15	5.38 ± 3.23
No	3.06 ± 2.69	4.55 ± 3.17	3.76 ± 2.95	3.80 ± 3.01
*t*	−1.445	1.033	1.469	3.651
*p*	0.149	0.305	0.142	**< 0.001**
Gravidas				
One pregnancy	3.47 ± 2.68	4.88 ± 3.04	4.34 ± 2.76	4.41 ± 2.89
Pregnancies ≥ 2	2.83 ± 2.67	4.48 ± 3.12	3.63 ± 3.03	3.80 ± 3.11
*t*	2.499	1.347	2.614	2.129
*p*	**0.013**	0.179	**0.009**	**0.034**
Mode of delivery				
Vaginal	3.11 ± 2.58	3.74 ± 2.90	3.54 ± 2.91	3.83 ± 2.89
Cesarean	2.82 ± 2.84	5.95 ± 2.92	4.27 ± 3.02	4.17 ± 3.32
*t*	1.220	−8.618	−2.793	−1.228
*p*	0.223	**< 0.001**	**0.005**	0.220
Status of experiencing challenges in breastfeeding (*n* = 525)				
Yes	4.16 ± 3.28	5.48 ± 3.12	4.55 ± 3.02	4.16 ± 3.37
No	2.94 ± 2.50	4.37 ± 3.06	3.65 ± 2.96	3.89 ± 3.01
*t*	3.174	2.985	2.510	0.706
*p*	**0.002**	**0.003**	**0.012**	0.481
Full compression of baby's mouth the areola (*n* = 525)				
Yes	2.79 ± 2.58	4.41 ± 3.13	3.56 ± 3.04	3.68 ± 3.01
No	4.42 ± 2.64	5.03 ± 2.90	4.68 ± 2.60	4.91 ± 3.11
*t*	−5.803	−1.936	−3.817	−3.735
*p*	**< 0.001**	0.054	**< 0.001**	**< 0.001**

*Note:* *F*: ANOVA test, post hoc Tukey test, SD: Standard deviation, and *t*: Independent groups *t*‐test were used. In the table, *p* values < 0.05 are shown in bold and the source of the difference between the groups is shown with symbols a, b and c.

In Model 1 explaining 11% of the variance with the regression analysis, the variables affecting the FAS level were detected as follows: employment outside the home (*p* = 0.008, confidence interval (CI) = −4.161 to −0.631), type of the latest delivery (*p* = 0.001, CI = 0.819 to 3.219), the status of experiencing challenges in breastfeeding (*p* = 0.014, CI = −3.840 to −0.431), and full compression of the baby's mouth to the areola (*p* = 0.030, CI = 0.166 to 3.322). In Model 2 accounting for 15% of the variance with the regression analysis, the factors affecting the HLSB‐II level were detected as age groups (*p* = 0.002, CI = 2.903 to 13.255), educational status (*p* = 0.041, CI = 0.154 to 7.054), employment outside the home (*p* < 0.001, CI = −22.498 to 9.758), and experiencing challenges during pregnancy (*p* = 0.013, CI = 2.167 to 18.607) (Table 3).

**TABLE 3:** Evaluation of variables affecting FAS and HLSBS levels of postpartum women through regression analysis.

	β	*t*	*p*	95% CI	
Model 1: The effects of sociodemographic and obstetric variables on FAS					
Employment outside the home	−2.396	−2.667	**0.008**	−4.161	−0.631
Monthly income of family	−0.101	−0.150	0.881	−1.426	1.224
Classification of BMI	−0.370	−0.933	0.351	−1.149	0.409
Smoking status	−1.335	−1.089	0.277	−3.744	1.074
Regular use of medications due to chronic diseases	−1.513	−1.116	0.265	−4.176	1.150
Number of deliveries	−0.821	−1.329	0.185	−2.036	0.393
Type of latest delivery	2.019	3.307	**0.001**	0.819	3.219
Status of experiencing challenges while breastfeeding	−2.136	−2.462	**0.014**	−3.840	−0.431
Lack of full compression of baby's mouth the areola	1.744	2.171	**0.030**	0.166	3.322
Accompanist with postpartum women	−0.453	−1.444	0.149	−1.070	0.164
*R*: 0.335, *R* ^2^: 0.113, Durbin‐Watson: 1.887 (***p* < 0.001**)					
Model 2: The effects of sociodemographic and obstetric variables on HLSBS					
Age groups	8.079	3.071	**0.002**	2.903	13.255
Educational status	3.604	2.055	**0.041**	0.154	7.054
Type of family	2.264	0.647	0.518	−4.622	9.150
Employment outside the home	−16.128	−4.981	**< 0.001**	−22.498	−9.758
Monthly income of family	1.608	0.589	0.556	−3.763	6.979
Status of experiencing challenges in pregnancy	10.387	2.486	**0.013**	2.167	18.607
Development of lacerations during delivery	2.962	1.293	0.197	−1.546	7.470
Status of breastfeeding	−2.187	−0.252	0.801	−19.280	14.905
*R*: 0.388, *R* ^2^: 0.150, Durbin‐Watson: 1.771 (***p* < 0.001**)					

*Note:* In the table, *p* values < 0.05 are shown in bold.

Abbreviations: CI, Confidence Interval; FAS, Fatigue Assessment Scale; HLSBS, Healthy Life Style Behavior Scale.

## 4 Discussion

This study evaluated the levels of physical challenges, fatigue, pain, and HLSB‐II experienced by women during the first 48 h of the postpartum period. In this study, the fatigue level of women employed outside of the home, having monthly higher income levels, with normal BMI classification, smoking, and using regular medication due to chronic diseases was found to be higher than that of those in the other groups. In contrast to these findings, one study found that women of lower socio‐economic status were disadvantaged in terms of fatigue (Giallo et al. 2015). In the study conducted by Kojima and Asazawa in 2020, women with pre‐pregnancy diseases within the early postpartum period were also determined to have high levels of fatigue (Kojima and Asazawa 2020). Symptoms of chronic disease or regular use of medication may contribute to increased fatigue in women. In addition, it is thought that women with a normal BMI classification may have experienced more fatigue because they had a greater ability to take responsibility for their health in the postpartum period. Furthermore, women with employment outside the home have been shown to experience problems such as fatigue and depression in the postpartum period, similar to this study (Anak Janting et al. 2023; Wilson et al. 2019). This may be due to the additional physical and psychological demands placed on women by employment outside the home. Future studies could be designed to eliminate these confounding factors or conduct research that takes these elements into account.

Multiparous women were found to experience lower levels of fatigue compared to primiparous women in this research. In a qualitative study conducted with primiparous pregnant women, it was stated that the women felt fatigued and weak in the category of physical changes (Çi̇çek Özdemi̇r et al. 2022). Contrary to these findings, several studies in the literature have emphasized that multiparous pregnant women have higher levels of fatigue than primiparous women (Cheng et al. 2015; Taylor and Johnson 2013). Primiparous women experience such situations as the transition to motherhood, breastfeeding, and baby care for the first time. However, experiences over birth and the postpartum process enable multiparous women to have more realistic expectations. Therefore, all of these factors may explain why primiparous women in this study felt more fatigued. Women giving a delivery through C‐section have higher fatigue levels than those having a vaginal delivery. After the deliveries through C‐sections, women may experience challenges such as the need for physical recovery and feeling pain at the incision site. For these reasons, the fatigue levels of women who had a C‐section delivery were higher than those who had a vaginal delivery (Lai et al. 2015; Xiao et al. 2020). As different from these findings, Çolak et al. revealed in a study in 2019 that the mode of delivery had no effects on women's fatigue levels (Çolak et al. 2019). Health programs should take into account that the mode of delivery in the postpartum period may affect women's fatigue levels.

In this study, the fatigue levels of the women experiencing challenges while breastfeeding, having the partner as an accompanist, and with lack of full compression of the baby's mouth were detected to be higher. In a study, fatigue levels were found to be lower among women not requiring assistance while breastfeeding (Senol et al. 2019). In some cultures, postpartum care is traditionally provided by experienced female family members, such as mothers or mothers‐in‐law, who may be more skilled in newborn care and household responsibilities. In contrast, partners, despite their emotional support, lacked practical knowledge and experience, which could lead to increased stress and fatigue for new mothers (El‐Morsey 2019). In the study carried out by Henderson et al. in 2019, it was determined that the women supported by their partners during the postpartum period were likely to experience fatigue at a higher rate (Henderson et al. 2019). Moreover, a study conducted in China found that women's postpartum depression levels were associated with fatigue and inadequate support from their mother‐in‐law (Yi and Ahn 2024). These cultural dynamics may explain why, in this study, women whose accompanists were their mothers or mothers‐in‐law reported lower fatigue levels compared to those supported by their partners. These findings suggest that the effectiveness of partner support in reducing postpartum fatigue may be influenced by cultural expectations and caregiving roles. Future studies could investigate how culturally specific postpartum care practices influence maternal well‐being and fatigue levels, and also examine the role of partners in postpartum recovery.

The pain perception varies, depending on the physical changes experienced by women in the postpartum period (Park and Bang 2022; Senol et al. 2019). In this study, the women with the first pregnancy, experiencing challenges while breastfeeding, and whose baby's mouth did not compress the areola fully were likely to have higher pain levels in the nipple and back than those in other groups. In a study conducted with mothers who gave birth prematurely, the pain in the nipple is seen as the most common physical challenge experienced by breastfeeding mothers (Park and Bang 2022). Proper breastfeeding practices may play a significant role in reducing physical pain in postpartum women. In this study, the women undergoing a C‐section had higher pain levels in incision site than those performing a vaginal delivery. Surgically induced pain may last longer than postpartum pain after vaginal delivery. In a study, however, the women who had vaginal deliveries were stated to have experienced more whole‐body pain and fatigue than those who had C‐section deliveries (Reghunath and Sangeetha 2019). In this study, the women with the first pregnancy, experiencing challenges in breastfeeding, and whose baby's mouth did not compress the areola fully were found to have higher waist pain. In similar studies, breastfeeding challenges were reported to be associated with pain in the nipple, back, and waist (Dave et al. 2022; Park and Bang 2022).

In this study, the mean score on the HLSB‐II scale was higher among women aged 31–49 years, from nuclear families, with higher education, employed outside the home, and with monthly income exceeding expenses, compared to those in other groups. In the study in which Makama et al. investigated the barriers to a healthy lifestyle in the postpartum period in 2021, the factors affecting a healthy lifestyle in the postpartum period were listed as lack of information, fatigue, physical and psychological health challenges, lack of social support, changes in priorities, and child care (Makama et al. 2021). The higher HLSB‐II scores among women with higher education, employment outside the home, and greater financial security suggest that socioeconomic status and education levels may play a crucial role in adopting healthier lifestyle behaviors. These findings highlight the need for targeted health promotion programs to support women with lower socioeconomic status in improving their health‐related behaviors. In the postpartum period, the HLSB‐II motivation of the mothers decreased due to such reasons as the shift of the focus of interest, especially with the birth of the baby and the mother's putting her health in the second place due to the needs of the baby (Christiansen et al. 2021; Makama et al. 2021). In this study, the HLSB‐II levels of the women experiencing challenges during pregnancy, those developing lacerations during delivery, and those breastfeeding their babies were found to be lower than those in the other groups. Factors such as pregnancy, events experienced during the birth process, and shifting the focus to the baby in the postpartum period may affect maternal health behaviors.

### 4.1 Limitations

Since the study was carried out in the postpartum service of a hospital located in the Central Anatolian region of blinded, these findings do not represent all puerperal women and cannot be generalized to the universe. The study was conducted within the first 48 h postpartum. The results of the study highlight the physical problems experienced by women in the early postpartum period and the variables affecting fatigue and HLSB‐II levels. Future studies could explore a longer postpartum period to provide a more comprehensive understanding of the ongoing physical and behavioral changes. In addition, during the data collection process of the study, breaks were allowed for the mother's personal needs, such as breastfeeding the baby and going to the restroom. The participants then completed the whole questionnaire, which may have given the mothers an opportunity to reflect on the topic of the study. One of the strengths of this study was the large sample size (*n* = 548), which increased the reliability of the results. In addition, the study sample varied in terms of variables such as number of pregnancies, number of deliveries, and mode of delivery. Taking these parameters into account allowed comparisons to be made between the large sample groups. The HLSB‐II results reflect the last trimester of pregnancy, as they capture established habits and routines that may influence postpartum adaptation. However, they do not provide insights into lifestyle behaviors in the later postpartum period. Future studies could focus on evaluating HLSB‐II outcomes in the later stages of the postpartum period to gain a more comprehensive understanding.

## 5 Conclusion

The study examined the physical challenges, fatigue, and HLSB‐II levels experienced by women during the first 48 h of the postpartum period. It was found that the fatigue levels of the women are affected by such variables as employment outside the home, having a C‐section delivery, experiencing challenges in breastfeeding, and lack of full compression of baby's mouth on the areola. Additionally, women who were accompanied by their partner reported higher levels of fatigue. The HLSB‐II levels of the women between 31 and 49 years of age, graduating from college, having a profession outside the home, and experiencing no problems of pregnancy were found to be higher than those in other groups. Considering the age groups, the women between the ages of 31 and 49 were seen to have higher pain levels on the incision site, back, and low back, compared to those in other groups. Additionally, the women experiencing their first pregnancy had higher pain levels in the nipple, back, and low back, compared to those with two or more pregnancies. The women giving birth through C‐section were determined to have more pain on the incision site and back than those having vaginal deliveries. The women experiencing challenges while breastfeeding had higher pain levels in the nipples, incision sites, and back than those experiencing no challenges in breastfeeding.

### 5.1 Relevance of Clinical Practice

Considering the study findings, the fatigue and pain levels of those having C‐sections and experiencing challenges in breastfeeding increase. In the clinics, it is necessary to determine the risk factors likely to affect women's fatigue and pain levels and to plan the care given by healthcare professionals (nurses, midwives, etc.). One of the most striking findings of the study is that women accompanied by their partners reported higher fatigue levels. Participation of the partners in antenatal training classes to prepare for prenatal parenting the baby care may provide effective support in the postpartum period. The study also found that HLBS‐II levels are associated with sociodemographic factors such as age, education, and employment status. For this reason, women should be trained on developing healthy behaviors during pregnancy. By addressing the physical, psychological and social aspects of women's health in the postpartum period, healthcare professionals may provide more comprehensive support for women's recovery and improve their overall well‐being during this critical period. Encouraging healthy behaviors may significantly improve quality of life and reduce fatigue in postpartum women.

## Author Contributions

**Yasemin Erkal Aksoy:** conceptualization, investigation, methodology, validation, software, formal analysis, project administration, writing – review and editing, writing – original draft. **Bihter Akin:** conceptualization, investigation, funding acquisition, validation, methodology, writing – review and editing, writing – original draft. **Sema Dereli Yilmaz:** conceptualization, writing – review and editing, investigation, validation, methodology, formal analysis.

## Ethics Statement

Approval was obtained from the Ethics Committee for Non‐Interventional Clinical Investigations of the Faculty of Health Sciences at Selcuk University (Registration number and date: 2021/1773 and November 27, 2021). The approval was also obtained from the Directory of the Hospital of the Meram Medical Faculty at Necmettin Erbakan University on November 23, 2021, where the study was conducted (Registration number: E‐14567952‐900‐120 036). The women participating in the study were informed about the study design, and their verbal consent was also obtained. The study was carried out in accordance with the ethical standards established in the Declaration of Helsinki.

## Conflicts of Interest

The authors declare no conflicts of interest.

## Data Availability

The data that support the findings of this study are available from the corresponding author upon reasonable request.
